# Modeling crude oil pyrolysis process using advanced white-box and black-box machine learning techniques

**DOI:** 10.1038/s41598-023-49349-x

**Published:** 2023-12-19

**Authors:** Fahimeh Hadavimoghaddam, Alexei Rozhenko, Mohammad-Reza Mohammadi, Masoud Mostajeran Gortani, Peyman Pourafshary, Abdolhossein Hemmati-Sarapardeh

**Affiliations:** 1 Key Laboratory of Continental Shale Hydrocarbon Accumulation and Efficient Development, Ministry of Education, Northeast Petroleum University, Daqing, 163318 China; 2 Ufa State Petroleum Technological University, Ufa, 450064 Russia; 3 Plekhanov Russian University of Economics, Moscow, 117997 Russia; 4 Department of Petroleum Engineering, Shahid Bahonar University of Kerman, Kerman, Iran; 5 National Iranian Oil Company, Tehran, Iran; 6 School of Mining and Geosciences, Nazarbayev University, Astana, Kazakhstan; 7 State Key Laboratory of Petroleum Resources and Prospecting, China University of Petroleum (Beijing), Beijing, China

**Keywords:** Energy science and technology, Engineering, Mathematics and computing

## Abstract

Accurate prediction of fuel deposition during crude oil pyrolysis is pivotal for sustaining the combustion front and ensuring the effectiveness of in-situ combustion enhanced oil recovery (ISC EOR). Employing 2071 experimental TGA datasets from 13 diverse crude oil samples extracted from the literature, this study sought to precisely model crude oil pyrolysis. A suite of robust machine learning techniques, encompassing three black-box approaches (Categorical Gradient Boosting—CatBoost, Gaussian Process Regression—GPR, Extreme Gradient Boosting—XGBoost), and a white-box approach (Genetic Programming—GP), was employed to estimate crude oil residue at varying temperature intervals during TGA runs. Notably, the XGBoost model emerged as the most accurate, boasting a mean absolute percentage error (MAPE) of 0.7796% and a determination coefficient (R^2^) of 0.9999. Subsequently, the GPR, CatBoost, and GP models demonstrated commendable performance. The GP model, while displaying slightly higher error in comparison to the black-box models, yielded acceptable results and proved suitable for swift estimation of crude oil residue during pyrolysis. Furthermore, a sensitivity analysis was conducted to reveal the varying influence of input parameters on residual crude oil during pyrolysis. Among the inputs, temperature and asphaltenes were identified as the most influential factors in the crude oil pyrolysis process. Higher temperatures and oil °API gravity were associated with a negative impact, leading to a decrease in fuel deposition. On the other hand, increased values of asphaltenes, resins, and heating rates showed a positive impact, resulting in an increase in fuel deposition. These findings underscore the importance of precise modeling for fuel deposition during crude oil pyrolysis, offering insights that can significantly benefit ISC EOR practices.

## Introduction

In-situ combustion (ISC) is a challenging thermal enhanced oil recovery (EOR) technique, defined as the process of oil recovery by burning the heavy oil in reservoir^1^. In this technique, pure oxygen or oxygen-enriched gas is injected into the reservoir to combust a portion of the crudes. In other words, a portion of the oil-in-place is oxidized and utilized as fuel to generate heat^2^. The initiation of combustion front involves either artificial or spontaneous ignition of the oil. Artificial methods, such as gas/air burners, steam/hot fluid injection, or electric ignition, can be employed to ignite the oil deliberately. Alternatively, spontaneous ignition occurs at or near the injection well, often facilitated by downhole igniters^3^. As oxygen-enriched gas is continuously injected, the combustion front will propagate toward the production well. This causes a lot of heat to be released within the reservoir, reducing oil viscosity and achieving oil recovery. One of the prerequisites for ISC is fuel availability in the reservoir for the sustainability of the combustion front. The fuel served during ISC consists of carbonaceous residues (mainly coke) deposited around the combustion front as a result of thermal cracking, pyrolysis, and distillation of crude oil. Eventually, the recovery of unburned oil is enhanced due to displacement agents made by gases and heat released from combustion, along with changes in the physical and chemical properties of reservoir oil^1,4,5^. During ISC, oxidation and pyrolysis of hydrocarbons take place, which strongly affect the quantity and quality of the formed fuel required for the sustainability of the combustion front. Pyrolysis is a chemical reaction involving crude oil exposure to heat in lack of an oxidizing medium^5–7^. Pyrolysis, cracking, vapourization, condensation, and dehydrogenation may occur during ISC, which affects the physical and chemical properties of the carbonaceous residue and are important for oil production^5,7^.

Over time, thermogravimetry analysis (TGA) and differential thermogravimetry (DTG) techniques have been employed as investigative instruments for studying ISC processes^5,8^. Specifically, TGA can monitor weight changes during the combustion of fuels or residues, yet it is important to acknowledge that it does not claim complete simulation capability for such intricate phenomena during ISC. Ciajolo and Barbella^6^ investigated the oxidation and pyrolysis of several heavy oils and their fractions using DTG profiles. Low-temperature (< 400 °C) and high-temperature phases were found in the thermal behavior of fuel, which includes the volatilization of paraffinic and aromatic fractions in the first phase, and the pyrolysis of polar and asphaltene fractions causing a particulate carbon residue in the latter. Ranjbar and Pusch^9^ experimentally showed that heat transfer and transferability characteristics of the pyrolysis medium as well as the colloidal composition of oil (such as asphaltenes and resins) have a noticeable impact on the fuel formation and composition. In another study, Ranjbar^10^ showed that clay minerals existing in the matrix raise fuel deposition during the pyrolysis process and catalyze the oxidation of fuel. Kok^11^ studied differential scanning calorimeter (DCS) and TGA of two heavy crudes and showed that the heavier oil deposited larger quantities of residue/fuel after distillation was complete. Karacan and Kok^12^ analyzed the pyrolysis of crude oils and their fractions using TGA and DSC and showed that asphaltenes and resins respectively have the most contribution to coke formation. In another laboratory study, Kok and Karacan^13^ showed that as crude oils' °API decreases cracking activation energy increases. They also indicated two main mechanisms along with their temperature ranges for mass loss, which included thermal cracking and vis-breaking (400–600 °C) along with distillation (20–400 °C). Ambalae et al.^14^ experimentally indicated that asphaltenes have the largest role in the formation of coke (fuel) among other fractions of crude oil. Kok^15^ showed that the heating rate influenced the reaction region peak, intervals, and burn-out temperatures in TG-DTG experiments of crude oil combustion. Li et al.^16^ showed that pyrolyzed and oxidized cokes are the main types of coke in the ISC process, releasing more heat than crude oil under similar conditions. A lot of research has been done on the catalytic impact of different compounds on oxidation and pyrolysis of various crudes and cokes^17–22^. The kinetics of combustion and pyrolysis of crudes and their fractions and the deposited coke have been investigated in some studies^13,22–28^.

In the realm of crude oil pyrolysis and oxidation, despite extensive laboratory research, recent attention has shifted to modeling approaches, particularly through machine learning regression. This artificial intelligence technique proves valuable in understanding the complex relationships within crude oil pyrolysis and oxidation processes, given the multitude of influencing parameters in ISC. Rasouli et al.^29^ investigated the pyrolysis of six crudes and represented a multilayer perceptron model for predicting the crude oil residue on the basis of TGA with a 3.5% error. Norouzpour et al.^30^ modeled crude oil pyrolysis employing a radial basis function neural network based on TGA of six crudes with a 5.8% error. Mohammadi et al.^31^ collected TGA experimental data from nine crude oils’ oxidation and presented a model using a generalized regression neural network with an error of 2.3%. In another study, Mohammadi et al.^32^ modeled the pyrolysis of 11 crude oils based on TGA data by applying a cascade forward neural network with an error of 1.04%. Despite the existence of several models for predicting crude oil pyrolysis, gathering more experimental data and applying cutting-edge and robust black-box and white-box machine learning techniques have the potential to engender streamlined mathematical correlations, thereby yielding more precise intelligent models.

In this study, 2071 experimental TGA findings for 13 different crude oils are gathered from the literature in order to precisely represent crude oil pyrolysis, which is a crucial reaction in the ISC EOR process. Four robust machine learning techniques, including three black-box approaches (Categorical Gradient Boosting—CatBoost, Gaussian Process Regression—GPR, Extreme Gradient Boosting—XGBoost), and a white-box approach (Genetic Programming—GP), are used to model the residual mass of crude oils at various temperatures obtained from TGA. High-precision statistical and graphical error analyses are utilized to validate the developed models and mathematical correlation. Eventually, sensitivity analysis is carried out to reckon the relative effect of inputs on crude oil residue obtained during pyrolysis.

## Data gathering and preparation

In this study, 2071 experimental TGA findings related to 13 distinct crude oils were gathered from the literature^12,13,24,29,30,33–35^ in order to precisely represent crude oil pyrolysis, which is a crucial reaction in the ISC EOR process. The database used in this work is more comprehensive than the one used in Mohammadi et al.’s study^31^ (i.e. 2015 TGA data for 11 distinct crude oils). Since the kind of crude oil affects how it is pyrolyzed, a variety of crude oils with the characteristics mentioned in Table 1 were chosen to serve as input data for our models.

**Table 1:** Parameters of different crude oils utilized in the research.

Crude oil	1	2	3	4	5	6	7	8	9	10	11	12	13
Asphaltene (wt%)	4.73	5.42	10.71	12	18.95	3.25	12.8	1	9.66	30.32	29	20.25	38.67
Resin (wt%)	8.09	6.01	13.73	14	16.42	4.01	8.8	23.3	14.04	34.27	22	12.66	39.91
Heating rate (°C/min)	1,5,10	1,5,10	1,5,10	10	1,5,10	1,5,10	10	5,10,20	1,5,10	8	10	10	10
Oil °API gravity	20.83	21.87	18.67	26.12	13	30.39	20.5	14	20.26	5.5	14.95	35.17	11.72
References	^29,30^	^29,30^	^29,30^	^12,13^	^29,30^	^29,30^	^24^	^34^	^29,30^	^33^	^12,13^	^35^	^35^

**Figure 1:**
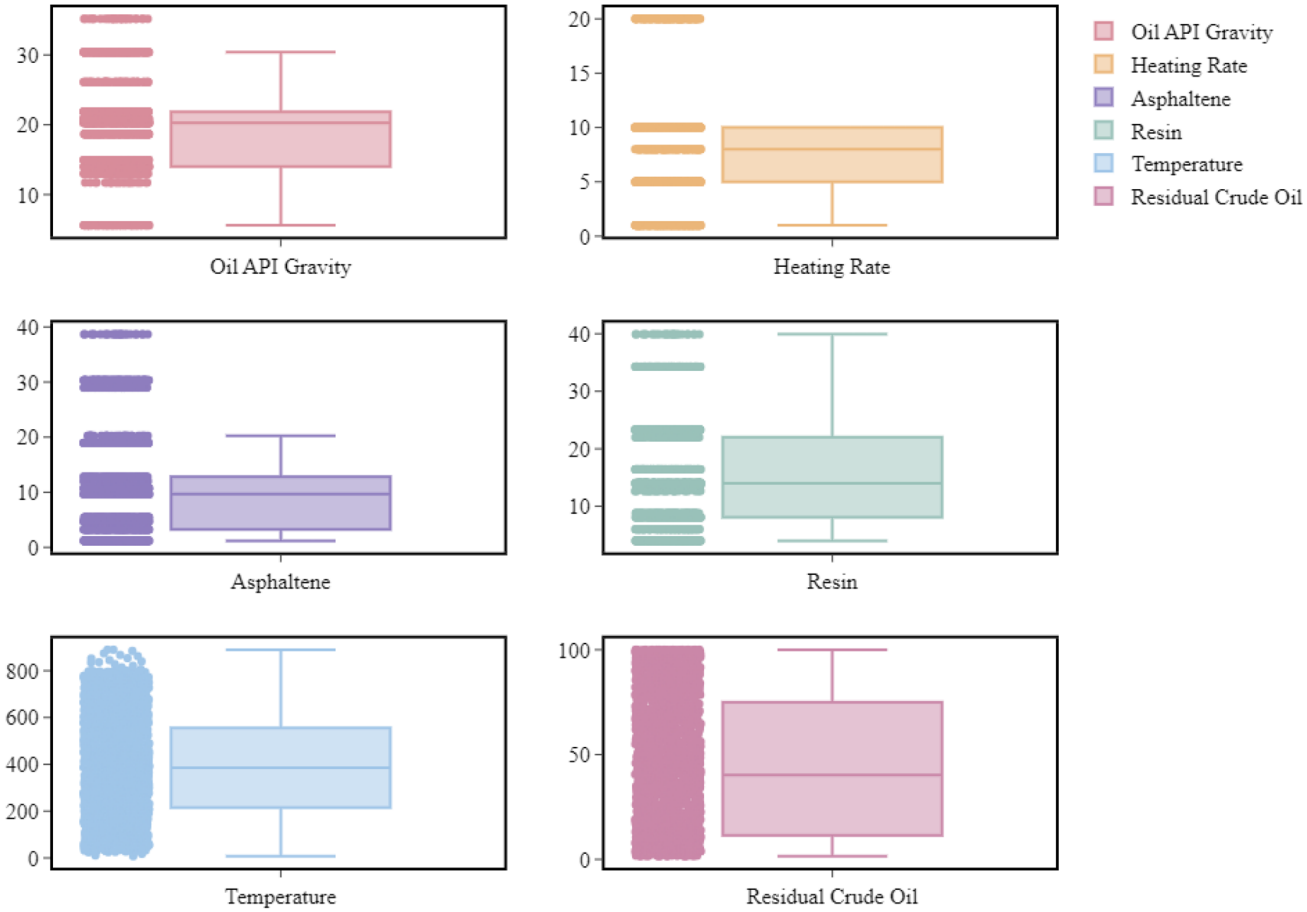
The box plots of parameters employed in the research.

For model training, factors identified in the literature as being important during the pyrolysis of crude oil^9,13,33,36,37^ were taken into consideration. In this research, the models' input parameters included the temperature, heating rate, weight percentage of asphaltenes and resins, and oil °API gravity. Since these values are often accessible, there is a large enough database for training the models. The model's result was the residual mass of crude oil at various temperatures. Table 1 lists the characterizations for crudes and heating rates utilized in this study's simulation. Additionally, Table 2 lists the output parameter and statistical descriptions of every model input variable, and Fig. 1 visually depicts the distribution of all the arguments.

**Table 2:** The statistical specifications of the input and target parameters of models.

Parameter	Maximum	Mean	Minimum	Median	Skewness	Mode	Kurtosis
Resin (wt%)	39.91	15.02	4.01	14.00	0.8222	23.30	0.3196
Asphaltene (wt%)	38.67	10.82	1.20	9.66	1.1170	1.20	0.4302
Temperature (°C)	889.71	390.65	7.50	385.71	0.1827	266.67	− 0.9631
Heating rate (°C/min)	20.00	7.07	1.00	8.00	0.6562	10.00	0.7317
Oil °API gravity	35.17	18.85	5.60	20.26	0.2091	14.00	0.0410
Residual crude oil (wt%)	100.00	44.54	1.52	40.22	0.2770	9.76	− 1.3832

Asymmetrical distribution, in contrast to symmetrical distribution, deviates from a regular and balanced pattern typically illustrated by a bell curve. Skewness may quantify the asymmetry of the distribution in this situation. Skewness value is positive when the probability function's left side contains the majority of the data, and vice versa. Conversely, kurtosis identifies the distribution shape in relation to the normal distribution. For instance, if the kurtosis is positive, it means that the normal distribution has a greater peak than the usual distribution^38^. According to the data in Table 2 and Fig. 1, the distribution and variation range of the input variables are broad enough to provide a generic model for forecasting the pyrolysis of crude oil. It should be mentioned that oil °API gravity, heating rate and especially asphaltene have a number of outliers which, in turn, definitely influence the precision of models. However, the vast majority of observations, as it is seen, are located within the box borders, making the impact of an error term insignificant. Despite the presence of outliers in the data, a thorough examination confirms their validity, indicating that they statistically differ from the majority of the data. As evident from the modeling results, these outliers do not significantly skew errors during the modeling process. As it is further observed, residual crude oil and temperature data are provided in a continuous form, and the distance between observations is insignificant, while other parameters including oil °API gravity, heating rate, asphaltene, and resin are represented with considerable gaps. Moreover, the median of the temperature and the residual crude oil used to develop models in this research are 385.71 and 40.22, respectively. The median of other parameters such as resin, asphaltene, heating rate, and oil °API gravity are 14, 9.66, 8, and 20.26, accordingly.

**Figure 2:**
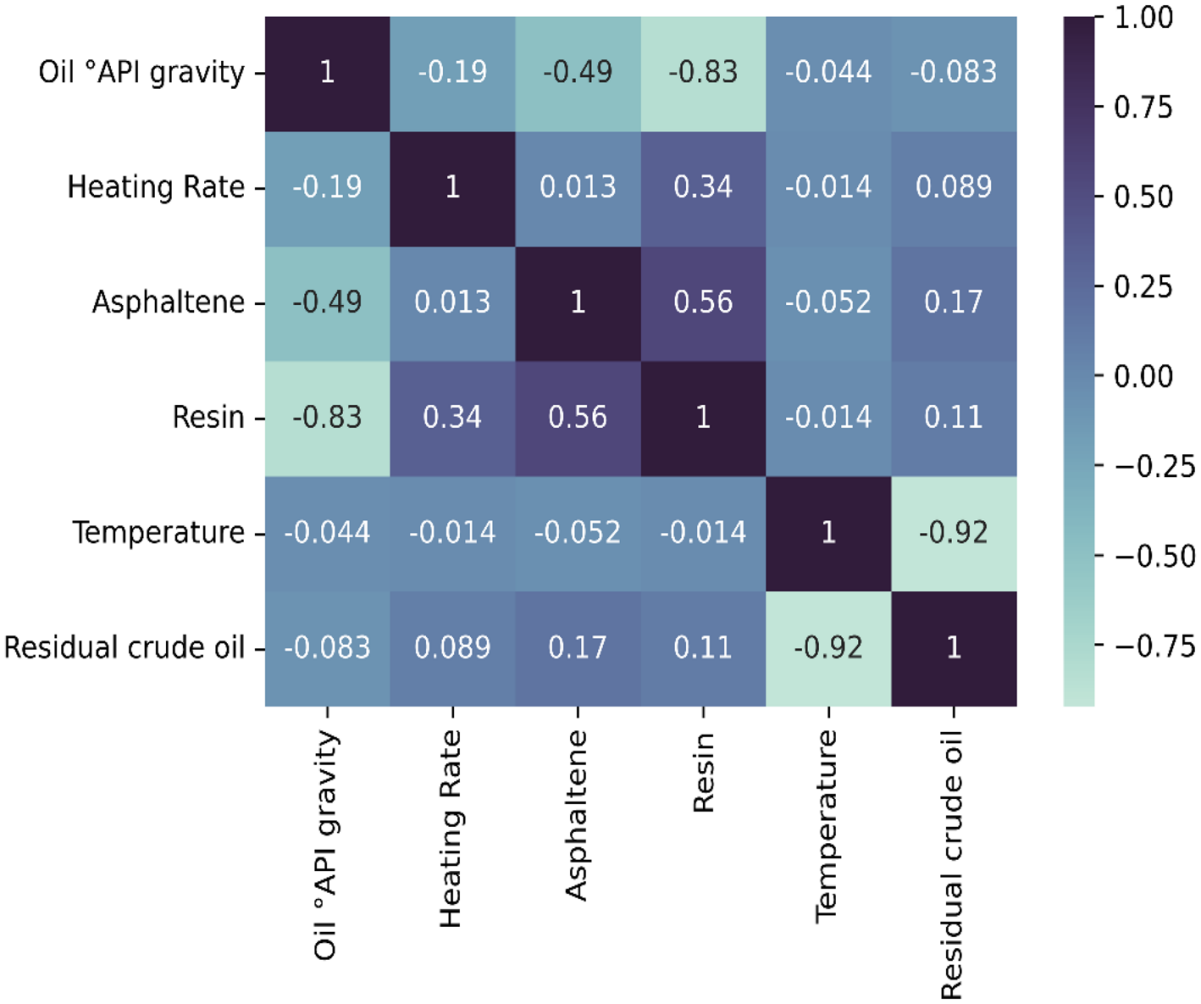
Correlation matrix of inputs and target data used in this study.

Figure 2 shows the correlation matrix of input data. As it is demonstrated, the temperature factor accounted for the greatest influence on mass estimate defining around 92% of its behavior. It should be stated that the correlation is negative, thus it means the bigger the temperature the less the mass value, and vice versa. Other parameters have a much smaller effect than the temperature on the target factor, but these parameters are essential for differentiation in crude oil characteristics and modeling of crude pyrolysis.

The dataset was partitioned into a training set comprising 80% of the total data and a test set with 20% randomly. Training involved using the training subset, while the test subset assessed the model's predictive performance. Here, K-fold cross-validation, specifically K-fold 6, was implemented to ensure each observation had an equal chance in training and validation. This involved randomly splitting the training data into 6 folds, fitting the model using 5 folds, and validating it with the remaining fold, a practice tailored to our dataset size.

## Model development

**Figure 3:**
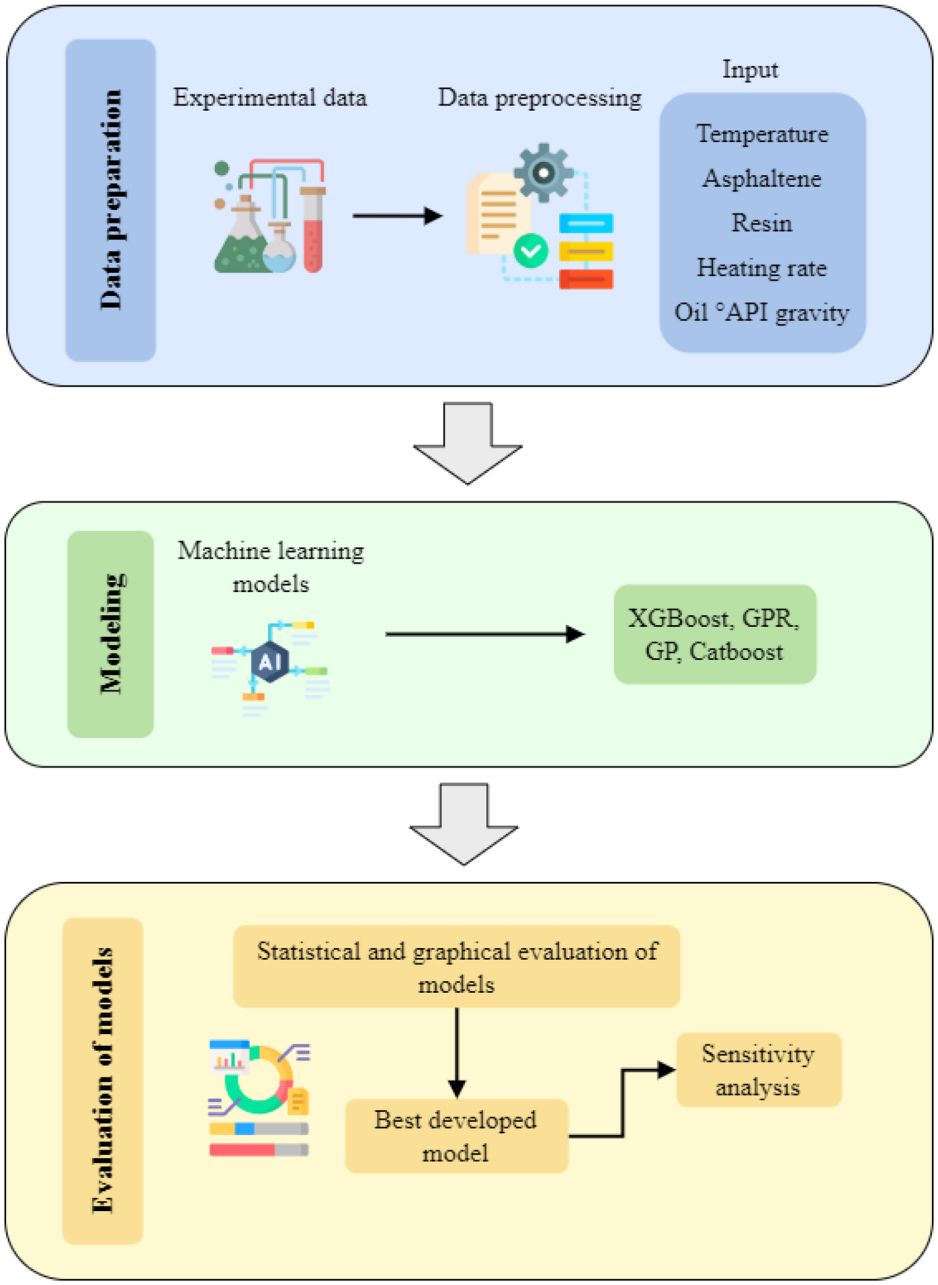
Flow chart of the research process in this work.

In this study, four different machine learning approaches were used for the purpose of the calculation of residual crude oil during pyrolysis. Among these techniques, one utilized was of white-box nature, and the others were of black-box origin. The flowchart represented below in Fig. 3 depicts the general schematic of the research showing the main steps of each stage employed.

### Gaussian process regression (GPR)

A common nonparametric modeling approach called GPR employs the Gaussian process before doing regression analysis^39^. It includes a prior Gaussian process solved using Bayesian inference as well as the regression residual.

A distribution over functions could be described by a Gaussian process, which is a group of random variables. Since a mean function $m(x)$ and a covariance function $k(x, x{\prime})$ may fully explain an actual process $f(x)$, it might be expressed as^40^:

1 $$f\left(x\right)\sim GP(m\left(x\right), k\left(x, { x}{\prime}\right))$$

The objective of GPR is to determine the mapping correlation between the input vector *x* and the observable y for a specific training dataset $D={\left\{{x}_{i}, {y}_{i} \right\}}_{i=1}^{n}$, where ${x}_{i}$ is the input vector of the *i*th sample and ${y}_{i}$ is the observation value of the *i*th sample^41^:

2 $$y=f\left(x\right)+ \zeta $$

where $\zeta$ is the additional disturbance that matches a Gaussian distribution with zero mean and variance ${\sigma }_{n}^{2}$. Calculating the covariance of noisy measurements *y* is as follows: $K\left(x, x\right)+ {\sigma }_{n}^{2}+{I}_{n}$, where $y={{[y}_{1 }, {y}_{2}, \dots ,{y}_{n} ]}^{T}$, $X={{[x}_{1 }, {x}_{2}, \dots ,{x}_{n} ]}^{T}$, *K* represents the covariance matrix, ${I}_{n}$ shows the *n*-dimensional identity matrix. Therefore, the joint distribution of the testing sample data ${x}_{*}$ under the prior may be computed as^41^:

3 $$\left[\begin{array}{c}y\\ f\left({x}_{*}\right)\end{array}\right]\sim N\left(0,\left[\begin{array}{cc}K(X,X)+{\sigma }_{n}^{2}{I}_{n}& K\left(X,{x}_{*}\right)\\ K\left({x}_{*},X\right)& K\left({x}_{*},{x}_{*}\right)\end{array}\right]\right)$$

According to Eq. (3), the mean of $f\left({x}_{*}\right)$ and covariance of $f\left({x}_{*}\right)$ may be written as^41^:

4 $$m\left(f\left({x}_{*}\right)\right)=E\left[f\left({x}_{*}\right)\mid X,y,{x}_{*}\right]=K\left({x}_{*},X\right){\left[K(X,X)+{\sigma }_{n}^{2}{I}_{n}\right]}^{-1}y$$

5 $${\text{cov}}\left(f\left({x}_{*}\right)\right)=K\left({x}_{*},{x}_{*}\right)-K\left({x}_{*},X\right){\left[K(X,X)+{\sigma }_{n}^{2}{I}_{n}\right]}^{-1}K\left(X,{x}_{*}\right)$$

In the traditional GPR (CGPR), the entire training dataset $D={\left\{{x}_{i}, {y}_{i} \right\}}_{i=1}^{n}$ is utilized to develop the nonparametric model and to compute the prediction findings for a specific test sample. The dimension of the covariance matrix *K* in CGPR is $n\times n$.

### Extreme gradient boosting (XGBoost)

Boosting applies to a family of learning techniques that increase the fit of ultimate models by mixing base models with basic functions^42^. The composite of basic models with fairly low precision^43^ creates a scalable solution that could identify deep interactions and is less susceptible to anomalies^44^. The gradient boosting approach, which consists of an effective linear model solver and a tree learning algorithm, is utilized to develop the model. Several objective functions, including regression, classification, and ranking, are supported by the boosting method. XGBoost, a free software package, delivers cutting-edge solutions to a variety of challenges, notably climate projections^45,46^. XGBoost with a scalable tree-boosting method performs more than 10 times quicker than current popular solutions on a single computer^44^. XGBoost includes several parameters, making it a complicated model. In addition, hyperparameters are required to limit the danger of over-fitting and forecast variability^47^. The number of iterations (n estimators) and the learning rate are the two key hyper-parameters that avoid overfitting in XGBoost. In this technique, n estimators relate to the complexity of the model; raising this parameter may result in a more robust model, but it might still overfit to a certain extent. The amount of iterations governs the degree of fit and so influences the optimal learning rate value, and conversely. Generalization effectiveness is often enhanced by minimizing the learning rate. Decreased learning rate may significantly enhance predictive accuracy^48^. The regularization term, proposed by Friedman^43^, assists users in avoiding overfitting and manages the model's complexity. Throughout the tuning procedure, model regularization factors such as lambda and alpha should be adjusted to the required regularization weight in order to improve the quality of the model.

### Categorical gradient boosting (CatBoost)

CatBoost is a machine learning technique founded on gradient boosting decision tree (GBDT) that was developed by Yandex researchers in 2017^49,50^. Through ranking promotion, it enhances GBDT, assures that all datasets may be utilized for training and learning, and decreases the over-fitting of training^51^. Due to its strong effectiveness, CatBoost has been employed in various sectors, notably driving style identification^52^ and diabetes diagnosis^53^. The traditional GBDT method substitutes the category feature with the average label value related to that category. In a decision tree, the mean label value is used as the segmentation criteria for nodes. This technique is referred to as greedy target-based statistics (greedy TBS) and is described as follows^49^:

6 $$\frac{\sum_{j=1}^{P} \left[{x}_{j,k}={x}_{i,k}\right]{Y}_{i}}{\sum_{j=1}^{n} \left[{x}_{j,k}={x}_{i,k}\right]}$$

In general, though, features include more data than lab. While the mean label value is employed to forcefully represent characteristics, conditional transfer takes place. The claim is that the supplied collection of findings ${\text{D}} = \{\text{X}_{\text{i}}, {\text{Y}}_{\text{i}},\}, \text{i} = 1,\ldots, \text{n}, \sigma=(\sigma_{1},\ldots,\sigma_{n} )$ is a permutation, and ${x}_{{p}_{A}k}$ may be replaced with^49^:

7 $$\frac{\sum_{j=1}^{P-1} \left[{x}_{{\sigma }_{j,k}}={x}_{{\sigma }_{p,k}}\right]{Y}_{{\sigma }_{j}}+aP}{\sum_{j=1}^{P-1} \left[{x}_{{\sigma }_{j,k}}={x}_{{\sigma }_{p,k}}\right]+a}$$

here, *P* is the a priori, and *a* is its weight (*a* > 0). The addition of a priori reduces the noise produced by the low-frequency category.

### Genetic programming (GP)

GP is a frequently used evolutionary method in evolutionary-based computing^54,55^. GP may be used to locate global optimum solutions in a wrapped search space. It may additionally generate optimization algorithms motivated by Darwin's theory of evolution^56^. GP employs an evolutionary path including selection, crossover, mutation, and cloning procedures to seek syntactic expressions that offer more connection between a set of independent (input) and dependent (output) elements^57^. GP is capable of optimizing model structure on its own, and its results are symbolic in nature. Moreover, its depiction is adaptable. These significant qualities make GP an excellent method for symbolic regression. GP-evolved solutions, in contrast, provide robust interpretability in terms of how features are learned or retrieved from the signals and how they influence categorization^58^.

### Model optimization and tuning

Optimal hyperparameter selection is crucial for algorithm performance. Tuning these parameters fine-tunes the model, significantly impacting accuracy and ensuring the algorithm is well-suited to the specific characteristics of the data, ultimately enhancing predictive capabilities^59^. In constructing each model and addressing overfitting, grid search was utilized to optimize the hyperparameters. The hyperparameters selected for each model differed, with their importance grounded in both theoretical principles and practical considerations. Table 3 provides a comprehensive overview of the selected hyperparameters for the algorithms implemented in this work.

**Table 3:** Optimal features for implemented models.

Model	Hyperparameter	Optimum value/feature
GPR	Kernel	Rational quadratic
	Length_scale	2
	Alpha	0.1
	Length_scale_bounds	(1e−05, 100,000.0)
GP	Generation Number	50
	Population size	8000
	Tree depth	10
XGBoost	n_estimators	100
	Learing_rate	0.1
	Subsample	0.9
	Max_depth	5
CatBoost	n_estimators	100
	Learing_rate	0.15
	Subsample	0.8
	Max_depth	5

## Evaluation of models

Utilizing seven statistical indicators, the accuracy of the suggested models was evaluated. The following metrics have been employed in the research: MAPE, SD, RMSE, R^2^, MAE, MBE, and NSE. The selection of such indicators is based on the fact that they are commonly considered to be the most representative and effective ones in the fields of statistics and machine learning. These are the descriptions for the measures listed below^60^:

Mean absolute percentage error (MAPE, %):

8 $${E}_{r}=\frac{1}{n}\sum_{i=1}^{n}abs(\left[\frac{{\left(y\right)}_{exp}-{\left(y\right)}_{pred}}{{y}_{exp}}\right])\times 100$$

Standard deviation (SD):

9 $${\text{SD}}=\sqrt{\frac{1}{n-1}\sum_{i=1}^{n}{\left(\frac{{y}_{exp}-{y}_{pred}}{{y}_{exp}}\right)}^{2}}$$

Root mean square error (RMSE):

10 $$RMSE=\sqrt{\frac{1}{n}\sum_{i=1}^{n}{({y}_{exp}-{y}_{pred})}^{2}}$$

Determination coefficient (R^2^):

11 $$ R^{2} = 1 - \frac{{\sum\limits_{i = 1}^{N} {(y_{\exp } - y_{pred} )^{2} } }}{{\sum\limits_{i = 1}^{N} {(y_{exp} - \overline{{y_{\exp } }} )^{2} } }} $$

where *N* shows the count of data, *y*_*exp*_ refers to the experimental data, and *y*_*pred*_ stands for predicted data by presented models.

Mean absolute error (MAE):

This estimate is a risk measure equivalent to the anticipated value of the absolute error loss or $l1$-norm loss. If $\hat{y}_{i}$ is the anticipated value of the *i*th sample and ${y}_{i}$ is the matching real value, then the calculated MAE over ${n}_{\text{samples}}$ is given by:

12 $$ {\text{MAE}}\left( {y,\hat{y}} \right) = \frac{1}{{n_{{{\text{samples}}}} }}\sum\limits_{i = 0}^{{n_{{{\text{samples}}}} - 1}} {\left| {y_{i} - \hat{y}_{i} } \right|.} $$

Mean bias error (MBE):

This parameter quantifies the average mistake in a forecast and is computed as:

13 $$MBE=\frac{1}{n}\sum_{i=1}^{n} \left({\tilde{y}}_{i}-{y}_{i}\right)$$

The Nash–Sutcliffe efficiency (NSE):

It is a normalized measurement that compares the residual variation (or "noise") to the variation of the observed data.

14 $$NSE=1-\frac{{\sum }_{t=1}^{T}{\left({y}_{o}^{t}-{y}_{m}^{t}\right)}^{2}}{{\sum }_{t=1}^{T}{\left({y}_{o}^{t}-{\overline{y} }_{o}\right)}^{2}}$$

Here, ${\overline{y} }_{o}$ represents the mean of observed data, while ${y}_{m}$ signifies the simulated data. Additionally, ${y}_{o}^{t}$ denotes the data being released at time instant *t.*

In combination with the statistical method, graphical analysis was utilized to verify the accuracy of the models. The following is a brief summary of what these graphical analyses imply^61^:

The plot of the error distribution is the percent relative error (*E*_*i*_), which is generated using the given equations and plotted against the experimental findings or variable. This graph illustrates the error pattern and the distribution of approximated *E*_*i*_ values along the axis of zero error.

15 $${E}_{i}=\left[\frac{{y}_{i,\mathit{exp}}- {y}_{i,pred}}{{y}_{i,exp}}\right]\times 100, i=\mathrm{1,2},3,\dots ..,n$$

The number of data units along the Y = X axis impacts the correctness of the model; the fewer points there are, the more effective the model.

A graph of cumulative frequency vs absolute relative error (*E*_*a*_) displays the accuracy of the model in anticipating any percentage of data. *E*_*a*_ is computed using the following equation:

16 $${E}_{a}=\left|\frac{{y}_{i,\mathit{exp}}- {y}_{i,pred}}{{y}_{i,exp}}\right|\times 100, i=\mathrm{1,2},3,\dots ..,n$$

## Results and discussion

### Developed correlation

For the GP algorithm, the following correlation which can accurately predict the target parameter was developed. In order to optimize the model, a thorough grid search was done to find the optimum population size, tree depth, tree length, maximum generations, etc. As a result, the comprehensible equation consisting of 3 input parameters and 10 additional coefficients was established.

17 $$\text{Residual Crude Oil} \, \left.=\left(\left(\left({c}_{0}-{c}_{1}\cdot \text{ Temperature}\right)\cdot {c}_{2}\cdot {\text{Temperature}}+\left({c}_{3}+{c}_{4}\cdot {\text{Temperature}}\right)\right)\cdot \left(\left({c}_{5}\cdot {\text{HeatingRate}}+{c}_{6}\cdot {\text{Asphaltene}}\right)-{c}_{7}\cdot {\text{Temperature}}\right)-{c}_{8}\cdot {\text{API}}\right)\cdot {c}_{9}+{c}_{10}\right)$$

$$\begin{aligned} {c}_{0}&=22.115\\  {c}_{1}&=0.030446\\  {c}_{2}&=0.002202\\  {c}_{3}&=18.099\\  {c}_{4}&=0.0057179\\  {c}_{5}&=3.2189\\  {c}_{6}&=2.8308\\  {c}_{7}&=1.1226\\  {c}_{8}&=1.9776\\  {c}_{9}&=0.0055491\\  {c}_{10}&=105.47\end{aligned}$$

**Figure 4:**
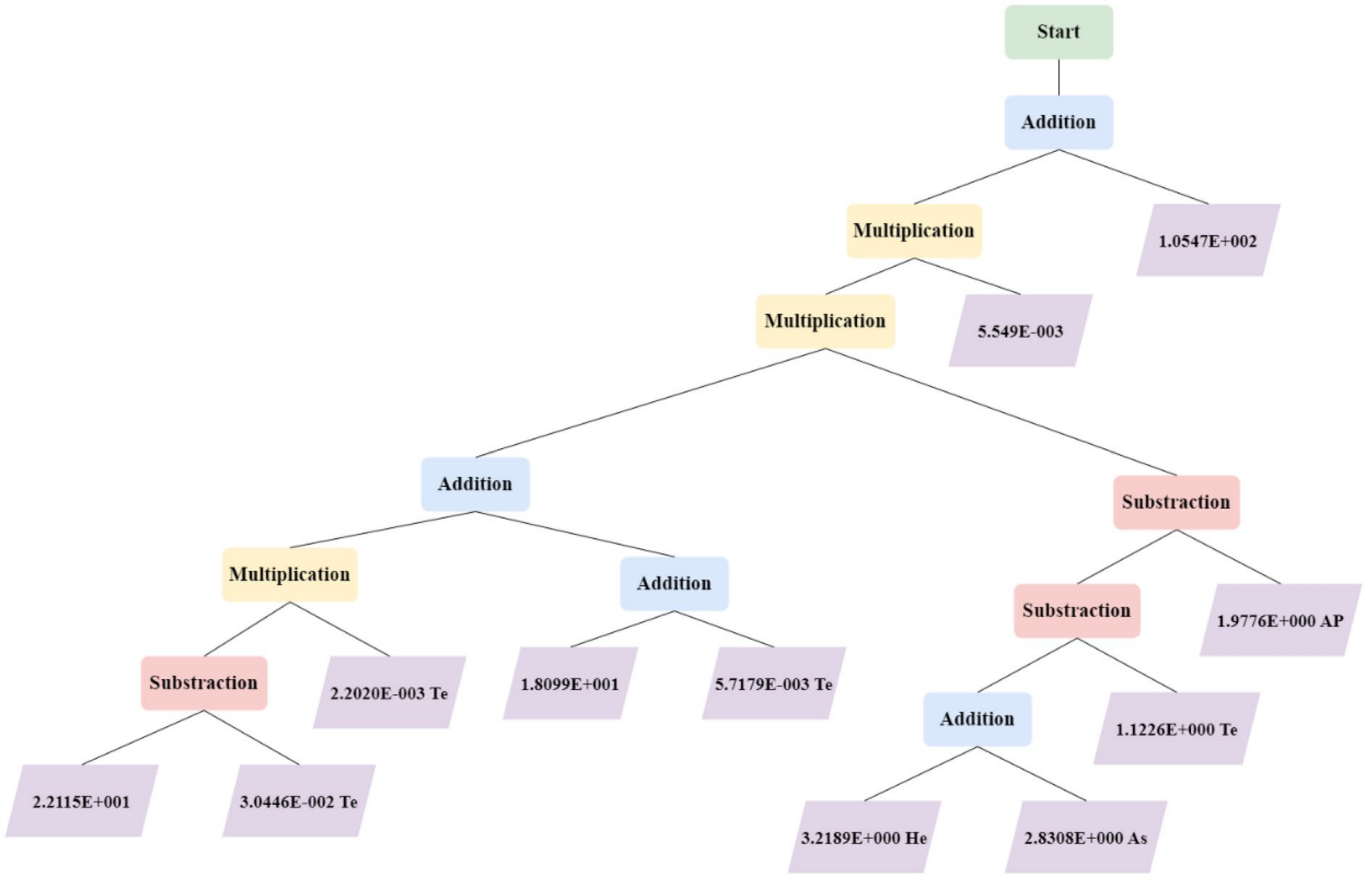
Schematic of the GP model developed in this study.

Figure 4 represents the schematic of the GP employed for estimating the residual crude oil during pyrolysis.

### Statistical evaluation of models

According to the statistical analysis provided in Table 4, the XGBoost model has provided the highest accuracy and reliability in terms of all the indicators. Its R^2^ and NSE are almost equal to 1, and the RMSE, SD, MAPE, MBE, and MAE are extremely small for the testing and training as well as the whole datasets. The GP approach has proved itself to be the worst among the four developed techniques, yet its precision is still quite high despite being less robust than others having 0.9820 of R^2^ for all portions of data. The middle positions are held by GPR and CatBoost. The performance of the first and the latter is rather decent with RMSE, SD, MBE, and MAE extremely close to zero and the estimate of R^2^ being more than 0.99. However, the GPR is better when comparing all the parameters except for MBE. All of these models outperform previously published models and correlations in terms of the precision of the forecasts. Summing up the statistical analysis, the following list from the best performance to the weakest can be established: XGBoost, GPR, CatBoost, and GP.

**Table 4:** The statistical errors of the proposed models in train, test, and total data sets.

Statistical criteria	RMSE	SD	R^2^	MAPE	MBE	MAE	NSE
CatBoost							
Test	0.61533	0.04181	0.99965	2.18740	− 0.00342	0.43954	0.99965
Train	0.50871	0.03915	0.99976	2.04320	0.00072	0.38990	0.99976
All	0.92566	0.05094	0.99919	2.76250	− 0.01995	0.63764	0.99919
GPR							
Test	0.42119	0.03885	0.99984	1.88670	0.00696	0.28109	0.99984
Train	0.42834	0.03992	0.99983	1.93660	0.00529	0.28755	0.99983
All	0.39137	0.03420	0.99986	1.68790	0.01364	0.25534	0.99986
GP							
Test	4.71424	0.15556	0.97950	10.38880	0.77854	3.37434	0.97915
Train	4.77625	0.15771	0.97885	10.55290	0.75432	3.42272	0.97849
All	4.45818	0.14665	0.98204	9.73420	0.87518	3.18126	0.98172
XGBoost							
Test	0.19878	0.01339	0.99996	0.70210	0.00195	0.14107	0.99996
Train	0.19382	0.01278	0.99997	0.68270	0.00214	0.13864	0.99997
All	0.21744	0.01559	0.99996	0.77960	0.00120	0.15073	0.99996

### Graphical evaluation of models

**Figure 5:**
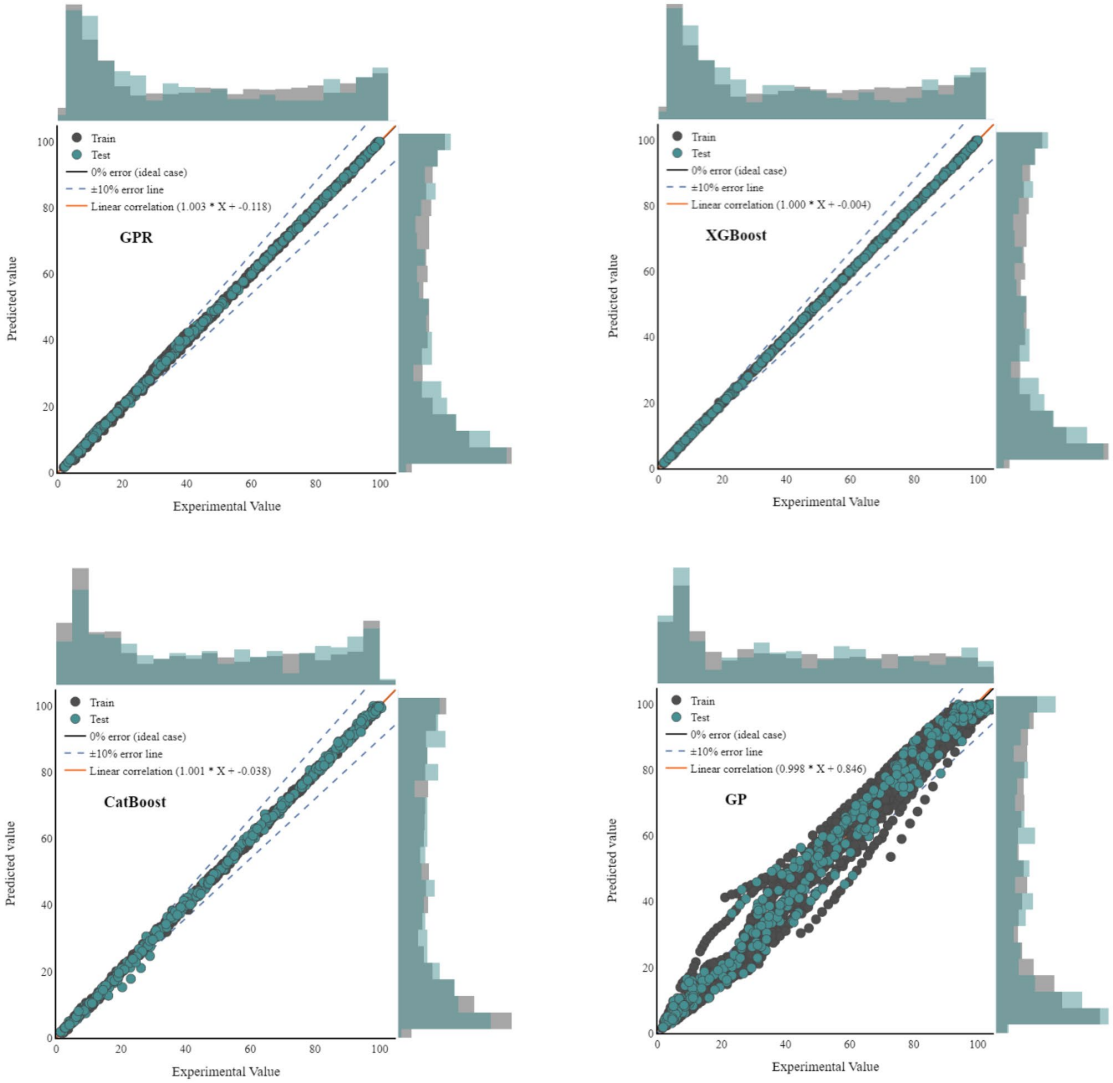
Cross plots for each of the testing and training datasets for target prediction.

In this regard, the graphical assessment of the models’ results was performed first by displaying the cross-plot of algorithms outcomes vs real data points, as shown in Fig. 5. Based on these diagrams, the spread of data points forms a line with a unit slope, indicating that the predicted and objective data points in all models except for GP are in excellent conformity. Having a unit slope though, XGBoost, GPR, and CatBoost differ from each other. As seen in the pictures, GPR and CatBoost have a number of insignificant outliers, whereas the XGBoost line is the smoothest among all.

**Figure 6:**
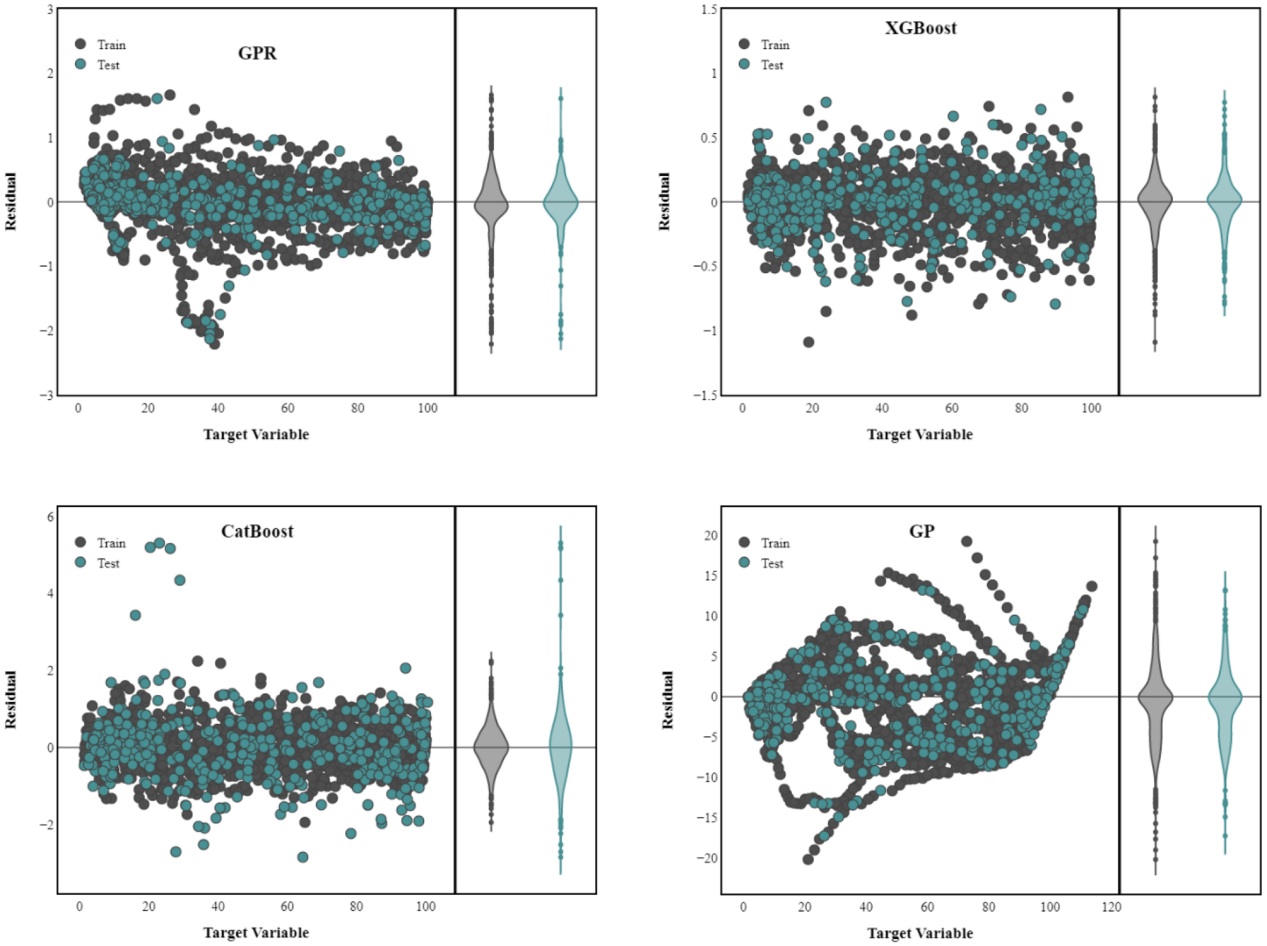
Residual plots for training and testing datasets.

A residual graph is a diagram depicting residuals along the vertical axis and the independent variable along the horizontal axis. The residual number is the discrepancy between the reported and expected numbers. According to the visual materials represented in Fig. 6, the best performance should be attributed to XGBoost possessing the smallest y-axis range (from approximately − 1 to around 1) and the lowest amount of outliers. GP is the least accurate with the spread of residuals from 20 to − 20. GPR and CatBoost are somewhat similar both having the same range in which the majority of observations are located, yet the outliers of CatBoost make it less precise than GPR.

**Figure 7:**
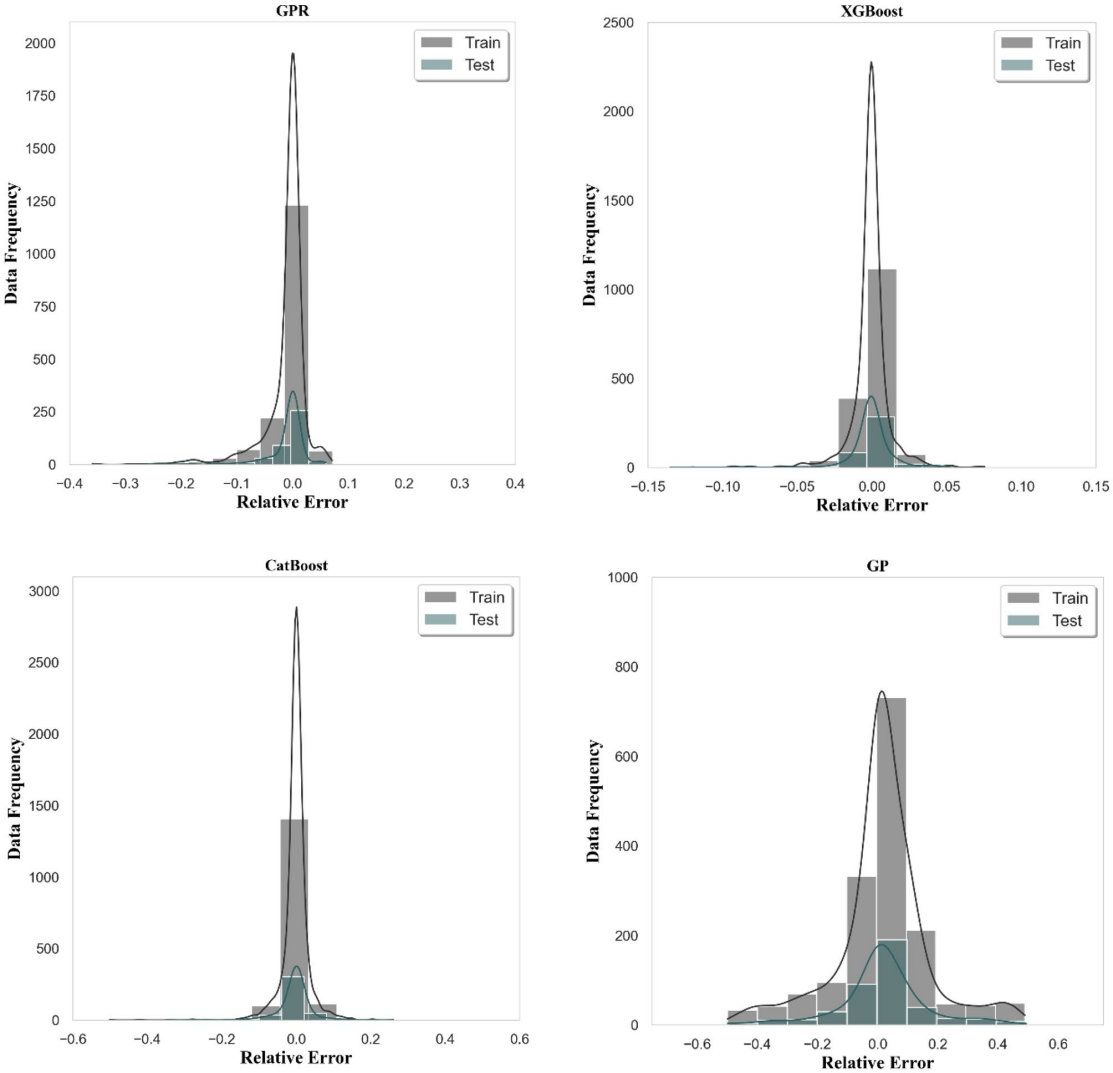
Histograms of error distribution for all developed algorithms.

A histogram of error distribution is an allocation of probabilities regarding a point projection that specifies the likelihood of each inaccuracy. Based on Fig. 7, the distributions are highly centered having little deviations in all approaches employed. The majority of the observations are at the point of zero relative error for both training and testing. However, the XGBoost is again the leader in the assessment as its relative error spread is the lowest one being from roughly − 0.14 to 0.08 for both training and testing.

**Figure 8:**
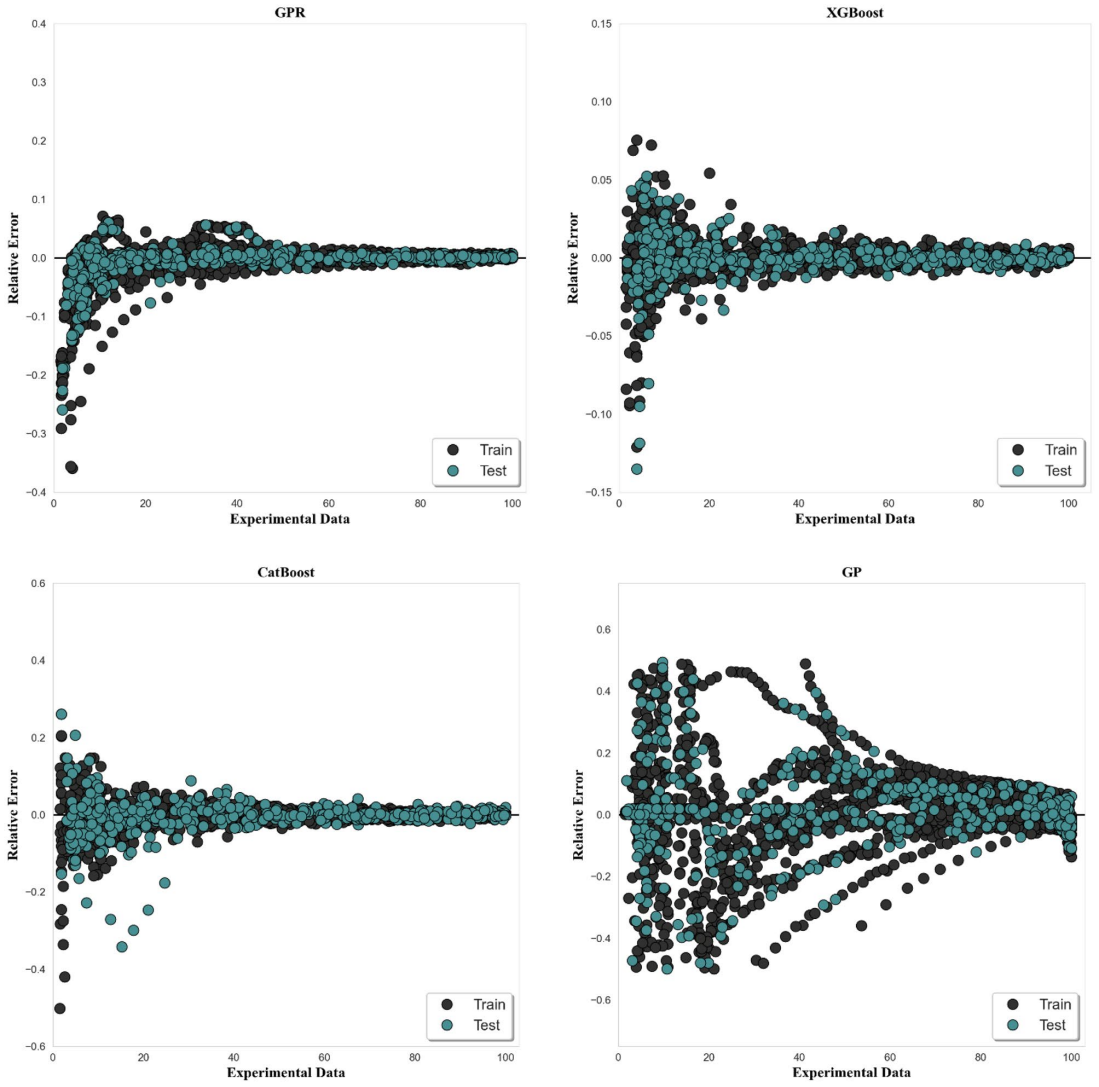
Relative deviations of predictions for all algorithms.

Figure 8 illustrates the relative deviation graph of the created models' results. The horizontal line of this graph represents experimental values, while the vertical axis represents the comparative deviation of model results from experimental values. This graph demonstrates that the comparative deviations of the suggested models are generally spread around the zero-deviation line, indicating that the models can predict the target data with tolerable error rates. As in all the cases, XGBoost effectiveness is the highest one comparing to other techniques utilized with the smallest relative error variation equal to around − 0.14 and 0.08. GP range is the greatest one being in the range from ~ − 0.5 to ~ 0.5 which indicated its lowest level of accuracy.

**Figure 9:**
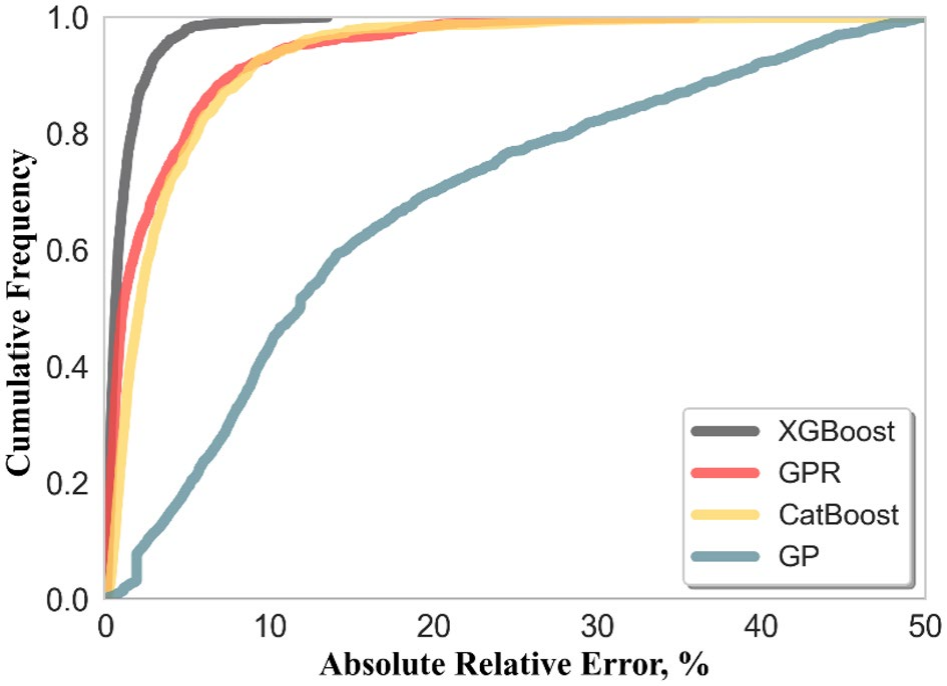
Cumulative frequency for all developed models.

A cumulative frequency is the total values distributed across multiple absolute relative error intervals. As depicted in Fig. 9, XGBoost, GPR, and CatBoost are the most effective methods for predicting the correct value of the target parameter, as the relative error of 90% of the data does not exceed 10–15%. The best precision is in the case of XGBoost, as the relative error of around 99% of the data is roughly equal to 5–7%. GP performance is worse than black-box models, as shown by the graph. Although the lower accuracy of the developed correlation is obvious and even predictable from the beginning, the advantage of correlation is fast prediction without the need for artificial intelligence-related knowledge, which is usually required to use black-box models.

In comparing this study with previous research, it can be asserted that a dataset comprising 2071 experimental TGA findings for 13 distinct crude oil samples was harnessed in this study, ensuring a comprehensive foundation for the modeling approach. This dataset represents the most extensive compilation utilized for modeling crude oil pyrolysis to date. The application of advanced machine learning techniques led to the development of models with high accuracy. Specifically, the XGBoost model achieved an overall MAPE of 0.7796% and an R^2^ of 0.9999, signifying a remarkable level of precision. This result compares favorably with prior investigations. Past studies in this domain have also sought to model crude oil pyrolysis and predict fuel deposition. Notably, Rasouli et al.^29^ developed a multilayer perceptron model with a 3.5% error for the pyrolysis of 6 crudes, Norouzpour et al.^30^, employed a radial basis function neural network with a 5.8% error for the pyrolysis of 6 crudes, and Mohammadi et al.^32^ utilized a generalized regression neural network with a 1.04% error for the pyrolysis of 11 crudes. While these models showcased respectable performance, our current study not only extends the dataset size but also harnesses a variety of machine learning techniques, enhancing accuracy and robustness in modeling. Furthermore, this study introduces a straightforward mathematical correlation that achieves remarkable accuracy with a mere 9.73% error. Formulating a coherent correlation between input and output datasets proves challenging in opaque methodologies. The application of black-box models demands sophisticated computer systems and specialized expertise, constraining widespread accessibility. Consequently, the development of user-friendly mathematical correlations using advanced white-box algorithms can streamline the prediction of fuel formation during crude oil pyrolysis, offering rapid and precise predictions without the necessity for specialized tools.

### Trend analysis

**Figure 10:**
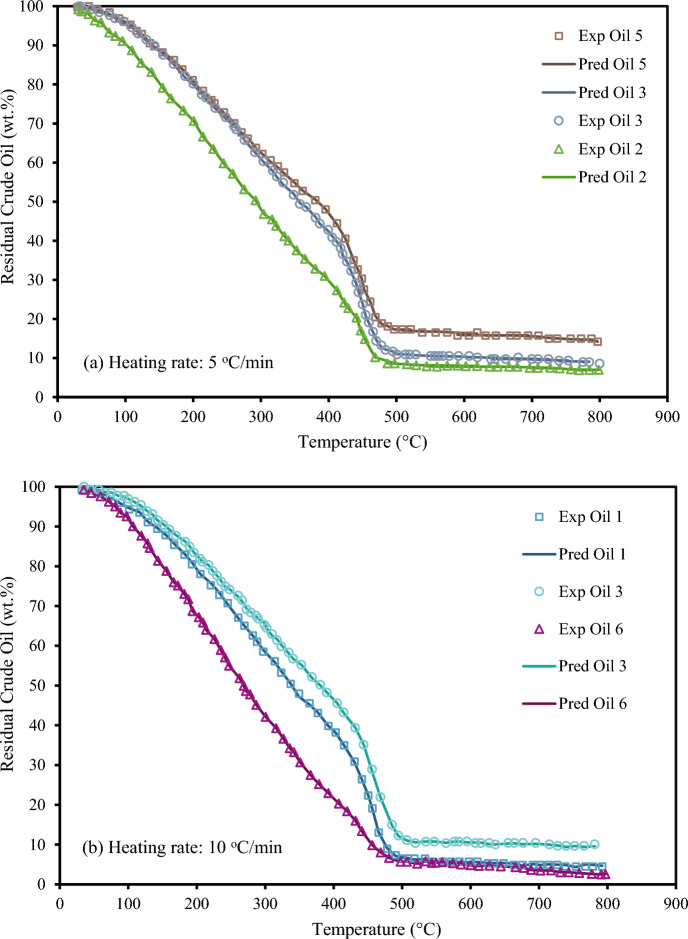
Experimental TGA data and XGBoost results for different crude oils.

**Figure 11:**
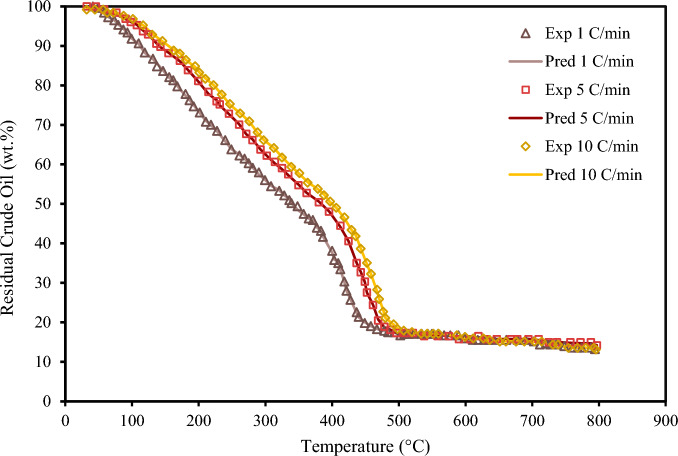
Experimental TGA data of crude oil #5 and XGBoost results for different heating rates.

**Figure 12:**
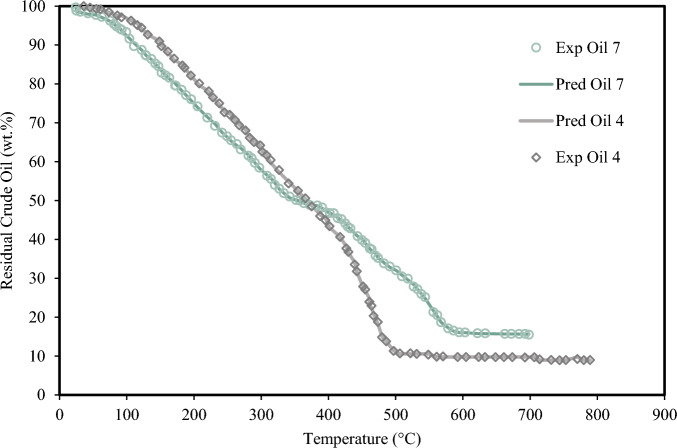
Experimental TGA data of crude oil #4 and #7 and XGBoost results in the heating rate of 10 °C/min.

Lastly, Figs. 10, 11 and 12 illustrate how the XGBoost model predicts residual crude oil during pyrolysis as a function of temperature for various crudes and heating rates. It should be mentioned that the chosen oil samples in each graph were connected to particular research to guarantee that the TG experimental settings were the same. Table 1 provides a summary of the heating rates and characteristics of these crude oils. The TG curves of several crudes (Oil 1, 2, 3, 5, and 6) with respect to temperature are shown in Fig. 10a,b. As shown in Fig. 10, the XGboost model successfully estimates the experimental trend for various heating rates and oils. Because crude oils have diverse constituents, so do their TG curves are likewise distinct. Heavy crudes often leave more residue because they contain more asphaltenes. In this instance, the suggested XGBoost model accurately recognizes the TG curve trend and forecasts the quantity of residue for each crude oil sample at various temperatures.

Figure 11 displays the TG curves for crude sample #5 at three diverse heating rates. As shown in Fig. 11, when the heating rate decreases, the crude oil's TG curve shifts to the left as a consequence of a longer exposure period to heat. The XGBoost model successfully predicts the experimental trend and tracks the influence of the heating rate.

Figure 12 displays the TG curves for crude samples #4 and #7 at the same heating rate, which is 10 °C/min. As shown in Fig. 12, the XGBoost model successfully predicts the experimental trend.

### Sensitivity analysis

**Figure 13:**
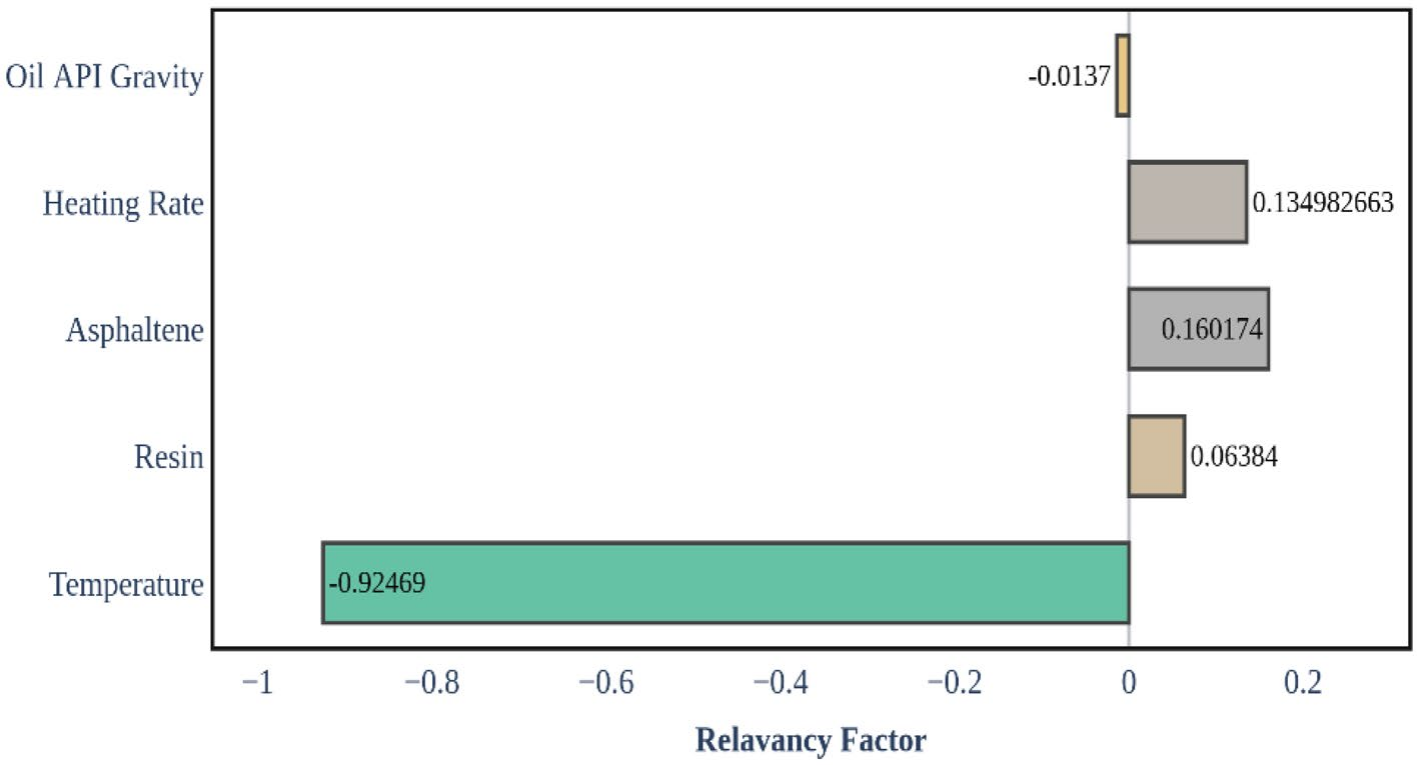
Sensitivity analysis using the XGBoost model response.

To assess the comparative significance of input variables on residual crude oil, the relevance factor (*r*) and the XGBoost model results are utilized. The accompanying method is utilized to calculate the *r* values for each input parameter^32,62^:

18 $$r\left(inp,\sigma \right)=\frac{\sum_{j=1}^{n}(in{p}_{i,j }-in{p}_{m,i})({\sigma }_{j}-{\sigma }_{m}) }{{\left(\sum_{j=1}^{n}{\left(in{p}_{i,j}-in{p}_{m,i}\right)}^{2}\sum_{j=1}^{n}{\left({\sigma }_{j}-{\sigma }_{m}\right)}^{2}\right)}^{0.5}}$$

where ${\sigma }_{m}$ is the average value of calculated residual crude oil and ${\sigma }_{j}$ is the *j*th value of assessed crude oil residue; and $in{p}_{i,j}$ and $in{p}_{m,i}$ are the *j*th and average value of the *i*th input parameter, correspondingly, where $in{p}_{i,j}$ are oil ^o^API gravity, heating rate, resins, asphaltenes, and temperature. Figure 13 depicts the relative effect and relevance of input parameters on residual crude oil. As it is seen, the most impact in the XGBoost model is attributed to the temperature with approximately − 0.92 significance. All other parameters such as resin, asphaltene, heating rate, and oil ^o^API gravity are not as influential as temperature having less than 0.16 of importance. Overall, among the mentioned inputs, temperature and asphaltenes owe the highest influence on the crude oil pyrolysis process. In addition, temperature and oil ^o^API gravity had negative impacts on fuel deposition, while asphaltenes, resins, and heating rates had a positive impact on fuel deposition during crude oil pyrolysis. This means that the higher the amount of asphaltene and resin of crude, the higher the amount of fuel (coke) formation.

The high negative impact of temperature on fuel deposition is attributed to the fundamental principles of pyrolysis. Elevated temperatures promote the thermal cracking and vaporization of hydrocarbons in crude oil, leading to a high reduction in the mass of residual crude oil. This behavior is consistent with the well-established pyrolysis process. Asphaltenes and resins are complex, high molecular weight components in crude oil. They tend to break down and contribute to coke formation during pyrolysis. Their positive impact on fuel deposition can be attributed to their transformation into solid carbonaceous residues, which enhance the overall fuel availability for sustaining the combustion front in ISC. Overall, an increase in asphaltene and resin content results in a reduction in mass loss during the pyrolysis of crude oil, consequently leading to increased fuel deposition. While heating rate is essential in governing the speed of temperature increase, its impact is relatively low in this model. With an escalation in heating rate, the TG curve for crude oil shifts to the right, signifying an increase in the mass of residual crude oil. The observed result is linked to the reduced exposure time of the crude oil to heat. Finally, Oil ^o^API gravity, with its lower significance, implies that its effect on fuel deposition is less pronounced. Typically, heavier crude oils characterized by lower ^o^API gravity tend to leave more residue, primarily due to a higher concentration of asphaltene. In summary, the technical reasons for these sensitivity analysis outcomes are rooted in the complex chemistry of crude oil pyrolysis. Understanding the behavior of these parameters can aid in optimizing ISC processes and improving the recovery of unburned oil.

## Conclusions

Crude oil pyrolysis analysis through TGA runs offers insights into fuel deposition during ISC EOR. This study aimed to precisely model crude oil pyrolysis by leveraging 2071 experimental TGA datasets obtained from literature sources. A suite of robust machine learning techniques, encompassing three black-box approaches (CatBoost, GPR, and XGBoost), and a white-box approach (GP), was employed to estimate crude oil residue at varying temperature intervals during TGA runs. Among the developed models and mathematical correlation, the XGBoost model exhibited exceptional precision, achieving an overall MAPE of 0.7796% and an R^2^ of 0.9999. Following the XGBoost model, GPR, CatBoost, and GP models provided the next best results, respectively. Notably, the GP model, despite displaying a slightly higher error compared to the black-box models, provided satisfactory results, making it a viable option for rapid estimation of crude oil residue during pyrolysis. Moreover, a sensitivity analysis was conducted to explore the relative impact and significance of inputs on residual crude oil during pyrolysis. Among these inputs, temperature and asphaltenes were identified as the most influential factors in the crude oil pyrolysis process. Higher temperatures and oil ^o^API gravity were associated with a negative impact, leading to a decrease in fuel deposition. On the other hand, increased values of asphaltenes, resins, and heating rates showed a positive impact, resulting in an increase in fuel deposition.

## Data Availability

The datasets used during the current study available from the corresponding author on reasonable request.
